# An evidence synthesis approach to estimating the incidence of symptomatic pertussis infection in the Netherlands, 2005–2011

**DOI:** 10.1186/s12879-015-1324-y

**Published:** 2015-12-29

**Authors:** Scott A. McDonald, Peter Teunis, Nicoline van der Maas, Sabine de Greeff, Hester de Melker, Mirjam E. Kretzschmar

**Affiliations:** Centre for Infectious Disease Control, National Institute for Public Health and the Environment, PO Box 1, 3720 BA Bilthoven, Netherlands; Julius Centre for Health Sciences and Primary Care, University Medical Centre Utrecht, PO Box 85500, 3508 GA Utrecht, Netherlands

**Keywords:** Pertussis, Symptomatic incidence, Bayesian evidence synthesis, Netherlands

## Abstract

**Background:**

Despite high vaccination coverage, infection with *Bordetella pertussis* is a current public health concern in the Netherlands and other European Union member states. Because surveillance data are subject to extensive under-ascertainment and under-reporting, incidence is difficult to determine. Our objective was to estimate the age-group specific incidence of symptomatic pertussis infection in the Netherlands over the period 2005–2011, using multi-parameter evidence synthesis.

**Methods:**

Age-specific seroconversion probabilities were estimated for 2007 using Netherlands population data stratified by age-group and cross-sectional population-wide serosurvey (PIENTER-2) data, with a sero-diagnostic cut-off of 125 EU/ml as a proxy for recent infection. Symptomatic probabilities were derived from a study of household contacts and from PIENTER-2. The annual number of symptomatic infected (SI) persons was estimated using evidence synthesis methods in a Bayesian framework, by combining the estimated incidence of infection with notification data and symptomatic probabilities.

**Results:**

An incidence rate of 128 SI cases per 10,000 population (95 % credible interval [CrI]: 110–150) was estimated for 2005, which decreased to 107 per 10,000 (95 % CrI: 91–126) for 2011. The degree of underestimation in statutory notified cases was age-dependent, ranging from 10-fold (10–19 years) to 69-fold (60+ years). The largest annual decreases in SI incidence rate over the study period were in the 1–4 and 5–9 years age-groups (24.3 %, 15.9 % per year, respectively).

**Conclusions:**

By synthesising all available data, the incidence of symptomatic pertussis and the extent to which SI is underrepresented by notification data can be estimated. Such estimates are essential for disease burden computation and for informing public health priority-setting.

**Electronic supplementary material:**

The online version of this article (doi:10.1186/s12879-015-1324-y) contains supplementary material, which is available to authorized users.

## Background

Infection with *Bordetella pertussis* is recognised as a current public health concern as it is endemic in the Netherlands and other European Union member states, despite relatively high vaccination coverage. The Dutch National Immunization Program has led to a high vaccination coverage for infants (~96 %) for more than 50 years. A two-fold rise in seroprevalence has been reported between 1995/6 and 2006/7 for persons aged >9 years, from 4.0 to 9.3 % [1], and an increasing trend in notified cases was reported over the period 1996–2012 [2]. For incidence estimation, notified and laboratory-confirmed case counts are of limited value due to extensive under-ascertainment and under-reporting. Indeed, pertussis surveillance data in most countries are inadequate for the accurate estimation of disease burden [3].

Mathematical modelling methods based on the kinetics of IgG-Ptx antibody titres [4] estimated the seroincidence of pertussis infection in the Netherlands to lie between 1 and 6 % per year [5]. Based on prevalence data from a population-wide serosurvey (PIENTER-1), the estimated incidence of infection in 1995/96 for persons aged 3–79 years was estimated at 6.6 % per year [6]. Comparison with the number of notified cases suggested the presence of 100-fold under-estimation of symptomatic infections. The results of similar methods applied to Danish data [7] indicate an even greater extent to which notification data under-estimate the number of infections. Although pertussis seroincidence is demonstrably high in the Netherlands, it is of considerable value to estimate the age-specific incidence of *symptomatic* infection to obtain an accurate picture of the current disease burden associated with pertussis infection, and of the variation in burden between age-groups.

Our goal was to estimate the unknown annual numbers of persons symptomatically infected (SI) with pertussis in the Netherlands in the period 2005–2011, and thus estimate the degree to which the incidence of SI is under-represented by notification data. Knowledge regarding these quantities will help inform decision-making regarding vaccination and other prevention initiatives [3]. In the Netherlands a number of indirect sources of data exist that may be useful for estimating SI. We employed multi-parameter evidence synthesis (MPES) to make optimal use of these available data. MPES is an established methodology for integrating various sources of data to estimate a quantity of interest for which there are no or limited direct data, and has been recently applied to estimating the prevalence or incidence of HIV, hepatitis C, and influenza virus infection [8–11].

## Methods

The study period chosen was 2005–2011. The start year of 2005 was selected because from the beginning of this year the acellular vaccine replaced the whole-cell vaccine administered to babies (the acellular vaccine as 4-years booster had been administered since 2001) [12].

### Data sources

Age-specific population data for the Netherlands for 2005 through 2011 were used to define the base population for each study year; these data were obtained from the website of Statistics Netherlands (http://statline.cbs.nl). We defined six age-groups: <1 year, 1–4 years, 5–9 years, 10–19 years, 20–59 years and 60+ years.

PIENTER-2, a cross-sectional seroprevalence survey conducted in the Netherlands in 2006/2007 provided age-group specific data on seroprevalence [13]. Based on a sero-diagnostic cut-off level for IgG pertussis toxin of 125 EU/ml [14], overall seroprevalence in the Netherlands general population was estimated at 3.4 % for 2006/2007 [1].

Since 1976 notification of pertussis to the Inspectorate of Health Care has been obligatory by law in the Netherlands. Notification data covering the study period were obtained from the Dutch online registration system for infectious diseases. The case definition for pertussis infection includes laboratory confirmation (or close contact with a person with laboratory-confirmed pertussis), and a clinical picture compatible with pertussis (i.e., serious cough with a duration of more than two weeks and/or coughing attacks and/or cough followed by vomiting).

To estimate the proportion of infected persons who are symptomatic, we used two data sources. First, the PIENTER-2 serosurvey [13] recorded the prevalence of coughing symptoms in the past year among infected individuals (defined according to a IgG-Ptx threshold of 62.5 EU/ml). Limited symptom data were available from PIENTER-2 for individuals aged 10 years and older (i.e., applicable to our 10–19, 20–59 and 60+ years age-groups only) [1]. These data were supplemented by data from the BINKI study of household contacts of pertussis-infected infants 6 months old or younger who had been hospitalised [15]. The BINKI study provided sufficient numbers of laboratory-confirmed infected contacts and the numbers reporting typical disease manifestation (defined as at least 2 weeks of coughing and one or more of the following: paroxysmal coughing, posttussive vomiting, inspiratory ‘whooping’), for the age-groups 1–4, 5–9, 10–19, and 20–59 years only.

As this modelling study used fully anonymised statutory notification and survey data, formal ethical approval from a medical ethical committee was not required.

### Evidence synthesis

We applied multi-parameter evidence synthesis to combine estimates of the incidence of infection (both symptomatic and asymptomatic) derived from the PIENTER-2 seroprevalence survey, annual notified pertussis case numbers, and the data on age-group specific symptomatic proportions, in order to estimate the critical model parameter: the number of SI cases per year, stratified by age-group. In this approach, annual numbers of SI cases are informed by indirect evidence from other model parameters for which observed data and/or prior information is available.

The evidence synthesis approach was a logical choice for addressing our research question, because SI incidence cannot be measured directly but can be inferred from other, existing data. A simple point estimate of SI incidence (i.e., SI = seroprevalence × symptomatic proportion) ignores information on the uncertainty associated with each component, and in the event that a component can be taken from different data sources/studies, the analyst is forced to either select a single source or to combine sources by averaging. The same multiplicative definition underlies our model; however, the evidence synthesis uses all the relevant data, allows several data sources for a given indicator to be integrated in a statistically sound way, and takes into account the uncertainty inherent in all data sources.

Estimation was conducted in a Bayesian framework, which is advantageous for model formulation and the easy incorporation of prior knowledge. The Bayesian framework ensures the correct propagation of uncertainty regarding model parameters (where ‘parameter’ includes subpopulation sizes, the proportion symptomatic, etc.), and permits any available prior information on these parameters to be flexibly combined with observed data (if available), to produce a posterior distribution. Uncertainty associated with a parameter value is expressed as 95 % credible intervals (CIs) around the median posterior estimates.

### Model specification

**Fig. 1:**
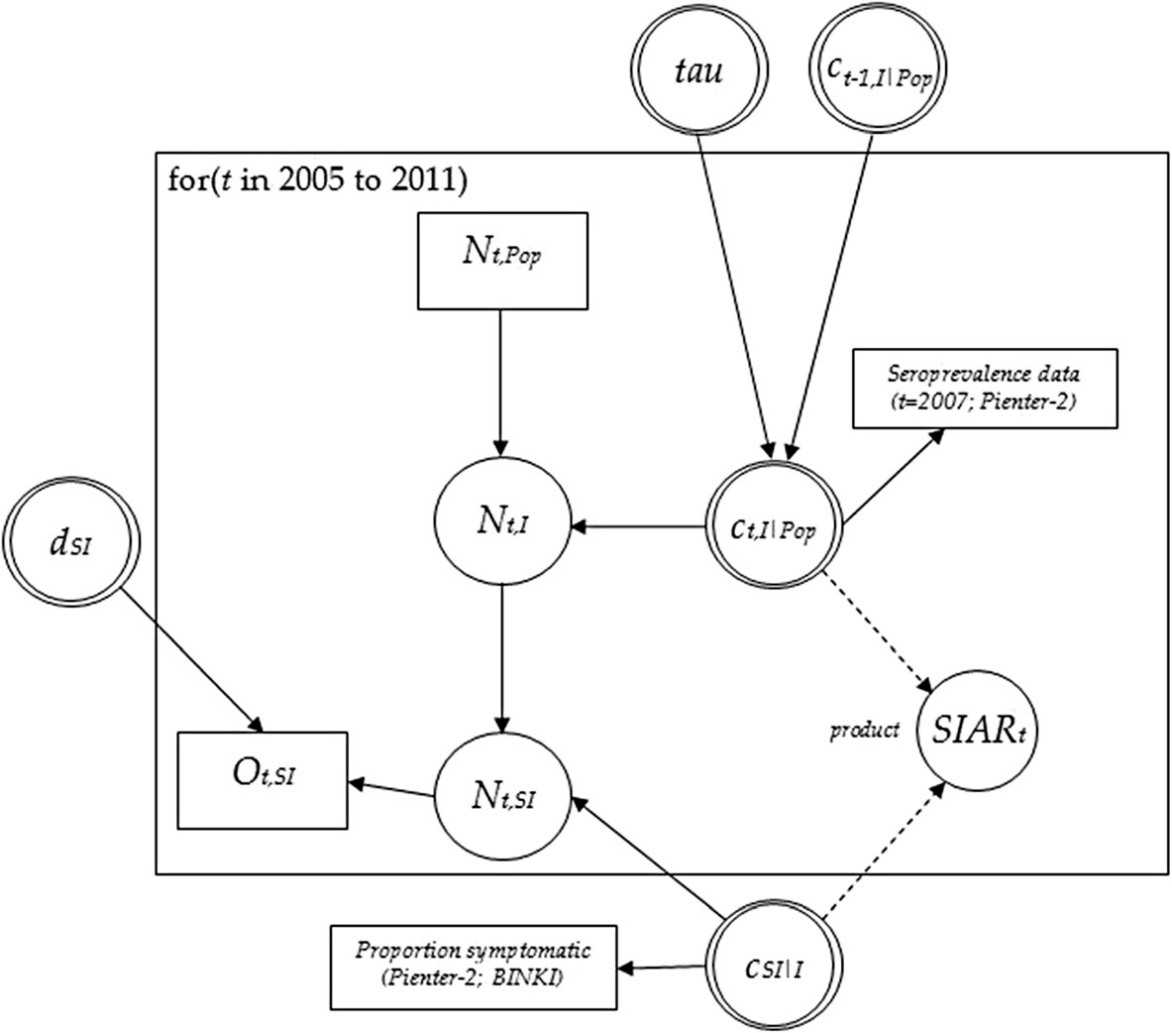
Directed acyclic graph of the relationship between model parameters and observed data; for clarity, only one age-group of the stratified model is shown. Distributional and functional relationships are indicated by solid and dashed lines, respectively. Circles indicate model parameters; double circles indicate parameters for which priors (either informative or vague) are applied. *N*_*t*_ and *O*_*t*_ refer to unobserved and observed numbers of individuals at time *t*, respectively; *c*_*t*_ are conditional probabilities, *SIAR*_*t*_ is the symptomatic infection attack rate, and *d* refers to detection probability

Figure 1 shows the relationship between the actual sizes of the subpopulations of interest, the observed numbers in each subpopulation (i.e., the true number of seroconversions per year, the number of pertussis cases with symptomatic infection), the conditional probabilities linking the true numbers corresponding to each subpopulation, and the sources of direct evidence (data) informing the model parameters.

We distinguish basic from functional parameters within an evidence synthesis model. Basic parameters can be assigned a prior distribution, whereas functional parameters are defined as functions of basic parameters. In the below, the notation *N*, *O*, and *c*_*a*|*b*_ indicate the actual number of persons in a subpopulation (the number to be estimated), the observed number, and a generic conditional probability of *a* given *b*, respectively. For instance, the subpopulation of individuals with evidence of pertussis infection (seroconversion) in a given year *t*, *N*_*t,S*_, is related to the total population, *N*_*t,Pop*_, by the conditional probability *c*_*t,S*|*Pop*_. In Fig. 1, the conditional probabilities *c*_*S*|*Pop*_ are informed by seroconversion data from PIENTER-2; the conditional probabilities *c*_*SI*|*I*_ are informed by data on coughing symptoms among infected persons, from PIENTER-2 and the BINKI study.

Parameters for six separate age-groups (<1 year, 1–4, 5–9, 10–19, 20–59, and 60+ years) were estimated whenever possible; i.e., if age-group specific data and/or prior information were available. The model computes the posterior distribution over all parameters and Table 1 lists all model parameters and the prior distributions adopted. The model specification ensures that the probability of seroconversion for a given age-group is correlated across the years of the study period. Through the links between subpopulations (i.e., *N*_*I*_ and *N*_*SI*_), variability in notified case numbers across years indirectly influences temporal variability in the seroconversion probability, *c*_*t,S*|*Pop*_ (and via a functional relation in which vaccination-related seroconversion is adjusted for, the temporal variation in infection probability). Intuitively, years with relatively high number of notified cases would be associated with a stronger infection pressure than years with fewer notified cases.

**Table 1:** Model parameters, the specified prior distribution or functional form, and evidence informing the parameter

Parameter	Description Distribution/functional form	Rationale	Evidence
*c*_*a,2007,S|Pop*_	Proportion of population that seroconverted in 2007	Vague prior on logit probability, assuming nothing is known about this parameter	Direct evidence from observed cases with IgG-Ptx titre above sero-diagnostic threshold, PIENTER 2 study
	Normal(0,1000)		
*c*_*a,t,S|Pop*_	Proportion of population that seroconverted each year	Random-walk prior assumed, in which seroconversion probability in current year is correlated with that in previous year	All model assumptions and data
	Normal(*c*_*a,t-1,S|Pop*_,τ_*a*_)		
*c*_*a,t,I|Pop*_	Proportion of population infected in each year	Adjustment for estimated proportion vaccination-related seroconversions	Based on modelling of longitudinal IgG-Ptx dataset
	*c*_*a,t,S|Pop*_ × (*1– propVaccRel*_*a*_)		
*c*_*a,SI|I*_	Proportion of infected population who are symptomatic	Informative priors, reflecting proportion of seroconversions with reported symptoms, PIENTER 2 study	Direct evidence from number of seropositive household members of infected infants with reported symptoms, BINKI study
	Separate Beta distributions for each age-group		
*d*_*a,SI*_	Proportion of true SI cases that are notified	Vague prior, assuming nothing is known about this parameter	All model assumptions and data
	Beta(1,1)		
*N*_*a,Pop*_	National population size	N/A	Population size estimates from Statistics Netherlands
	N/A		
*N*_*a,t,I*_	Size of infected population in each year	Functional relationship between the incidence and the national population size according to probability of seroconversion, PIENTER 2	All model assumptions and data
	*N*_*a,t,Pop*_ × *c*_*a,t,I|Pop*_		
*N*_*a,t,SI*_	Size of symptomatically infected population in each year	Functional relationship between the incidence of symptomatic infection and the number of incident infections according to symptomatic probability	Direct evidence from observed number of notified cases (binomial likelihood for ‘true’ number of SI cases with detection probability *d*_*a,SI*_)
	*N*_*a,t,I*_ × *c*_*a,SI|I*_		
*SIAR*_*a,t*_	Symptomatic infection attack rate	Symptomatic infection attack rate is defined as the product of component conditional probabilities	All model assumptions and data
	*c*_*a,SI|I*_ × *c*_*a,t,I|Pop*_		

#### Model parameters

Basic parameters are those model parameters to which a prior distribution is assigned (see Table 1), and include age-group dependent conditional probabilities and detection probabilities. The probability of seroconversion, *c*_*S*|*Pop*_, is also time-dependent, because the force of infection is assumed to vary across years.

*c*_*a,t,S|Pop*_*,* Probability of seroconversion in the population, for age-group *a* in year *t*

*c*_*a,SI|I*_*,* Probability of being symptomatic given infected, for age group *a*

*d*_*a,SI*_*,* Proportion of actual SI cases that are observed (i.e., notified), by age-group

The probability of infection in the population is functionally related to the probability of seroconversion, by adjusting for the proportion of seroconversions estimated to occur due to previous, recent vaccination (see Additional file 1). The proportion of vaccination-related seroconversions is zero for age-groups older than 1–4 years and 5–9 years, and zero for the <1 year age-group (under the simplifying assumption that the full series of infant vaccinations is required before a titre >125 EU/ml can be achieved; see Additional file 1):

$$ {c}_{a,t,I\Big| Pop}={c}_{a,t,S\Big| Pop}\times \left(1 - proportion\  vaccination- related\  seroconversions\right) $$

The following parameters relate the numbers of persons between subpopulations:

$$ \begin{array}{l}{N}_{a,t,I} = {N}_{a,t, Pop}\times {c}_{a,t,I\Big| Pop}\\ {}{N}_{a,t,SI} = {N}_{a,t,I}\times {c}_{a,SI\Big|I}\end{array} $$

The symptomatic infection attack rate (SIAR) is a functional parameter expressed as the product of age-group specific conditional probabilities:

$$ \mathrm{SIAR} = {c}_{a,SI\Big|I}\times {c}_{a,t,I\Big| Pop} $$

#### Relevant subpopulations and distributional assumptions

The subpopulations of interest are *N*_*I*_ and *N*_*SI*_ (Fig. 1). Because *N*_*SI*_ cannot be observed, the **‘**detection probability’ parameter *d*_*SI*_ relates the observed values (i.e., number of notified cases) to the true, or actual numbers (see below).

### Infected (both symptomatic and asymptomatic)

The probability of seroconversion was informed by cross-sectional seroprevalence survey data from 2006/7 (PIENTER-2) and a pre-determined sero-diagnostic cut-off level of IgG pertussis toxin. A titre of >125 EU/ml is a highly specific indicator of recent infection (within the previous 6 months) [14]; thus a titre exceeding this threshold served as a proxy for seroincidence [1]. Because of a lack of direct data for all years of the period modelled – data on this parameter were only available from the PIENTER-2 serosurvey carried out from February 2006 until June 2007 [13] – this data source was assumed to inform the year 2007 only. Data on age-group specific number of seroconversions (*y*) and number of persons tested (*n*) were therefore used directly to inform the prior distribution for the year 2007:

$$ {y}_{a,2007,I} \sim Binomial\left({n}_{a,2007,I},{c}_{a,2007,I\Big| Pop}\right) $$

### Symptomatic infected

The number of notified (observed) SI cases, *O*_a,SI_, was assumed to be binomially distributed, given the true (unobserved) number of symptomatic infections, *N*_a,SI_. The detection probability, *d*_*a,SI*_, was given a vague Beta prior distribution since it is unknown. The detection probability varied by age-group but was invariant across years; this encodes the belief that the likelihood of notification is constant over time, and entails that variability in notified cases between years drives variability in the actual number of symptomatic infections.

$$ {O}_{a,t,SI} \sim Binomial\left({N}_{a,t,SI},{d}_{a,SI}\right) $$

The conditional probability of exhibiting symptoms given infection with pertussis was estimated as time-independent (this parameter was assumed to be biological, and so not be affected by study year), but was stratified by age-group. This parameter was informed by two studies. The first study was based on data from the PIENTER-2 serosurvey conducted in 2005/6 [1]. We constructed Beta priors from the reported prevalence of coughing in the past year among persons with presumptive infection (defined using a 62.5 EU/ml threshold). For the age-groups 10–19 and 20–59, the coughing prevalence was 24 and 22 %, respectively. We extrapolated the value of 36 % (95 % CI: 27–44 %) cited for persons aged 65–79 years in this study to the 60+ years age-group*.* For age-groups under 10 years, we specified vague Beta priors as no data were available.

Second, for the age groups 1–4, 5–9, 10–19, and 20–59 years, we used data from the BINKI study of household contacts of pertussis-infected infants 6 months old or younger who had been admitted to hospital [15] to update the Beta priors. Overall, 159 out of 299 household contacts with laboratory-confirmed infection were symptomatic; the age-group specific symptomatic proportions were available for four age-groups: 32/56 (1–4 years), 22/39 (5–9 years), 17/25 (10–19 years) and 87/176 (20–59 years) (obtained from original data; S. de Greeff, pers. comm). Given that there was only a single <1 year-old contact in the BINKI study, a symptomatic proportion of 60 % was adopted for this age-group (N. van der Maas, pers. comm.) with a wide 95 % confidence interval of 40–80 % assumed.

$$ {c}_{a,SI\Big|I} \sim Beta\left({\alpha}_a,{\beta}_a\right) $$

#### Correlation in seroconversion probability across time

Variability and dependence in the force of infection across time were represented by specifying random-walk priors for the probability of seroconversion, *c*_*a,t,S|Pop*_ (see Fig. 1). The probability of seroconversion for a given age-group, *c*_*a,t,S|Pop*_, was allowed to vary across time but to be correlated with the probability of seroconversion in previous year(s), through specification of a random-walk prior for this parameter. The precision of the (logit of the) probability of seroconversion parameter was assigned the vague prior distribution Gamma(0.001,0.001). The random-walk prior on seroconversion probability, by “borrowing strength” across time, effectively allows for autocorrelation in seroconversion prevalence across successive years.

$$ \begin{array}{l} logit\left({c}_{a,t,S\Big| Pop}\right) \sim Normal\left(0,\ 10000\right)\mathrm{f}\mathrm{o}\mathrm{r}\;t=2005\\ {} logit\left({c}_{a,t,S\Big| Pop}\right) \sim Normal\left( logit\left({c}_{a,t-1,S\Big| Pop}\right),{\sigma^2}_a\right)\kern0.72em \mathrm{f}\mathrm{o}\mathrm{r}\ t=2006\dots 2011\\ {}{\tau}_a = 1/\ {\sigma^2}_a \sim Gamma\left(0.001,\ 0.001\right)\end{array} $$

Thus, the posterior probability *c*_*a,t,S|Pop*_ for each age-group *a* could vary between years, but only to the extent that is determined by prior assumptions and controlled by annual variation in the total notifications. Because of the chain of relationships specified between the estimated number of seroconversions, *N*_*a,t,S*_, and the observed case data informing *N*_*a,t,SI*_ (see Fig. 1), the number of notifed cases in a given year influences the posteior probability of seroconversion for that year.

#### Model inference

For each parameter, sampling of the posterior distributions was carried out via Markov-chain Monte-Carlo methods using OpenBUGS version 3.2.1 [16] and the BRugs package [17] for the R statistical programming environment [18]. BUGS code is provided in Additional file 1. Two independent chains were run for 230,000 iterations, with the first 150,000 iterations treated as burn-in and discarded. Brooks-Gelman-Rubin diagnostic plots were checked to establish that convergence of the chains was satisfactorily achieved.

The presence and magnitude of temporal trends in posterior median estimated SI incidence rates were evaluated using Poisson regression, also using R.

## Results

**Fig. 2:**
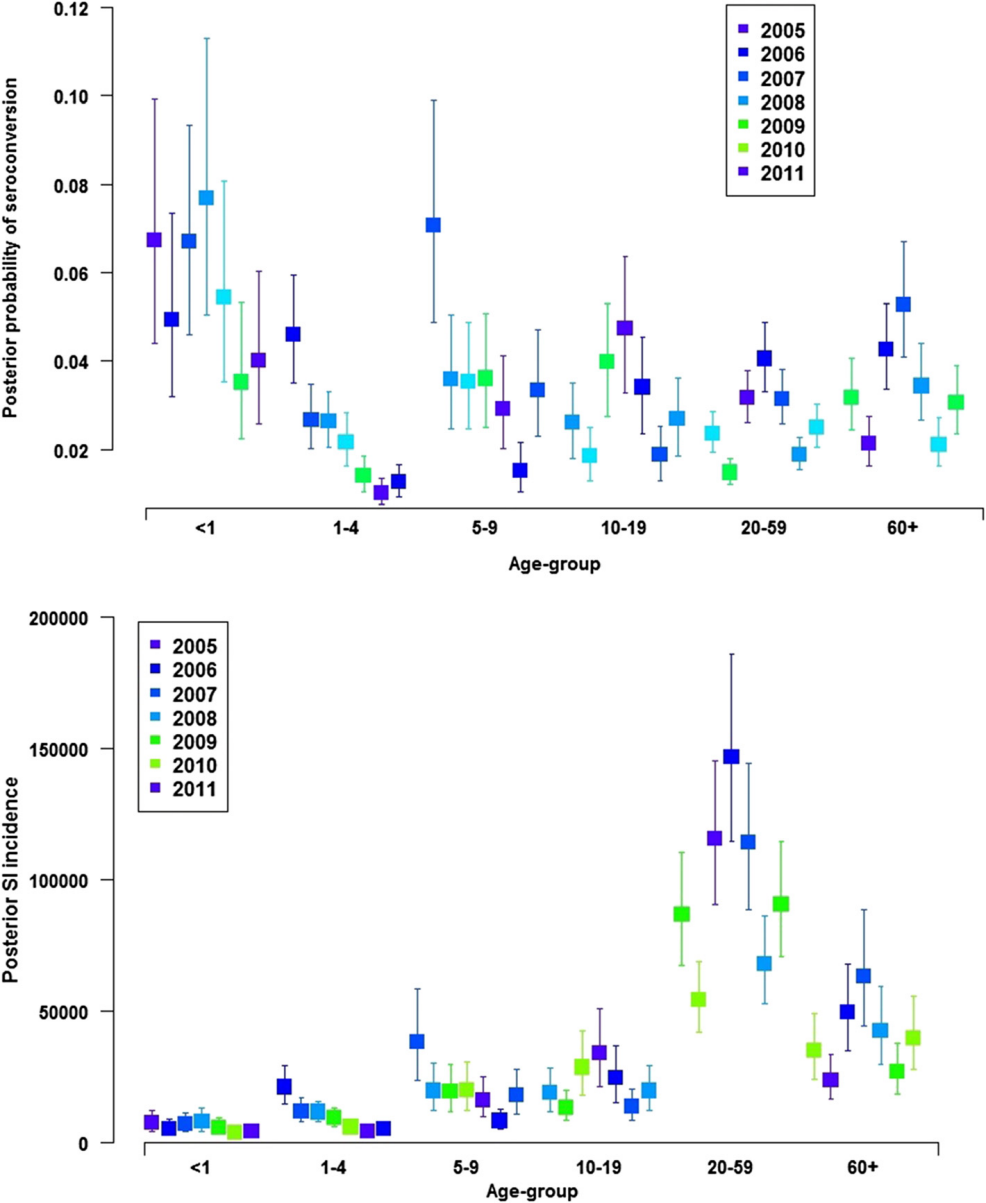
Posterior probability of infection (upper panel) and estimated posterior incidence of symptomatic pertussis infection (lower panel), shown separately by age-group and calendar year. Capped segments indicate 95 % credible intervals

Tables 2 and 3 show, for each year of the study period, the posterior summaries of the parameters of interest, and the estimated incidence rates for each subpopulation, aggregating over all age-groups. Both the estimated overall incidence of infection (i.e., *N*_*t*,*I*_, including both symptomatic and asymptomatic cases) and the overall incidence of SI varied considerably between 2005 and 2011. Peak numbers of 703,000 (95 % CrI: 617,200–794,500) infected persons and 283,300 (241,000–332,800) SI cases were estimated for 2008. The posterior probability of infection and SI incidence varied by age-group and across time (Fig. 2). The extent of uncertainty in the age-group and year- specific estimates for these parameters means that apparent differences within a particular age-group or year should only be interpreted considering the precision of the estimates (Fig. 2). We note that age-group variation within a given year is related to variation in seroconversion probabilities, and temporal variation for a given age-group is related to temporal variation in notified cases.

**Table 2:** Estimates of the probabilities of infection and symptomatic infection

	All age-groups		10–19 years		20–59 years	
Year	*c*_*I|Pop*_	SIAR	*c*_*I|Pop*_	SIAR	*c*_*I|Pop*_	SIAR
2005	0.030	1.3 %	0.026	1.0 %	0.024	0.9 %
	(0.027–0.034)	(1.1–1.5)	(0.018–0.036)	(0.6–1.4)	(0.019–0.028)	(0.7–1.2)
2006	0.019	0.8 %	0.019	0.7	0.015	0.6 %
	(0.017–0.021)	(0.7–0.9)	(0.013–0.026)	(0.4–1.0)	(0.012–0.018)	(0.5–0.8)
2007	0.035	1.4 %	0.040	1.4 %	0.032	1.3 %
	(0.031–0.040)	(1.2–1.7)	(0.028–0.054)	(0.9–2.2)	(0.026–0.038)	(1.0–1.6)
2008	0.043	1.7 %	0.047	1.7 %	0.041	1.6 %
	(0.038–0.049)	(1.5–2.0)	(0.033–0.064)	(1.1–2.6)	(0.033–0.049)	(1.3–2.1)
2009	0.032	1.3 %	0.034	1.2 %	0.032	1.3 %
	(0.028–0.036)	(1.1–1.5)	(0.024–0.046)	(0.8–1.8)	(0.026–0.038)	(1.0–1.6)
2010	0.019	0.8 %	0.019	0.7 %	0.019	0.8 %
	(0.019–0.022)	(0.6–0.9)	(0.013–0.025)	(0.4–1.0)	(0.015–0.023)	(0.6–1.0)
2011	0.026	1.1 %	0.027	1.0 %	0.025	1.0 %
	(0.023–0.030)	(0.9–1.3)	(0.019–0.036)	(0.6–1.5)	(0.020–0.030)	(0.8–1.3)

Posterior median estimates of the probability of infection, *c*_*I|Pop*_, and the symptomatic infection attack rate (SIAR), for all years in the study period, shown aggregated over all age-groups and for the 10–19 years and 20–59 years age-groups. 95 % credible intervals are in parentheses

**Table 3:** Estimated symptomatic infection incidence rates and number of cases over the study period

Year	*Rate*_*SI*_ (95 % CrI)	*N*_*SI*_ (95 % CrI)
2005	128	209100
	(110–150)	(178700–244100)
2006	79	129300
	(68–92)	(110700–150700)
2007	142	233000
	(122–166)	(199600–271500)
2008	173	283300
	(147–203)	(241000–332800)
2009	128	210400
	(108–150)	(178500–246600)
2010	76	125600
	(64–89)	(106500–148000)
2011	107	178600
	(91–126)	(151700–210000)

Posterior median estimates of the incidence rates (per 10,000 persons) (*Rate*_*SI*_) and number of cases of symptomatic infection (*N*_*SI*_) with pertussis (aggregated over all age-groups), for the years 2005 through 2011. 95 % credible intervals (CrI) are in parentheses

**Fig. 3:**
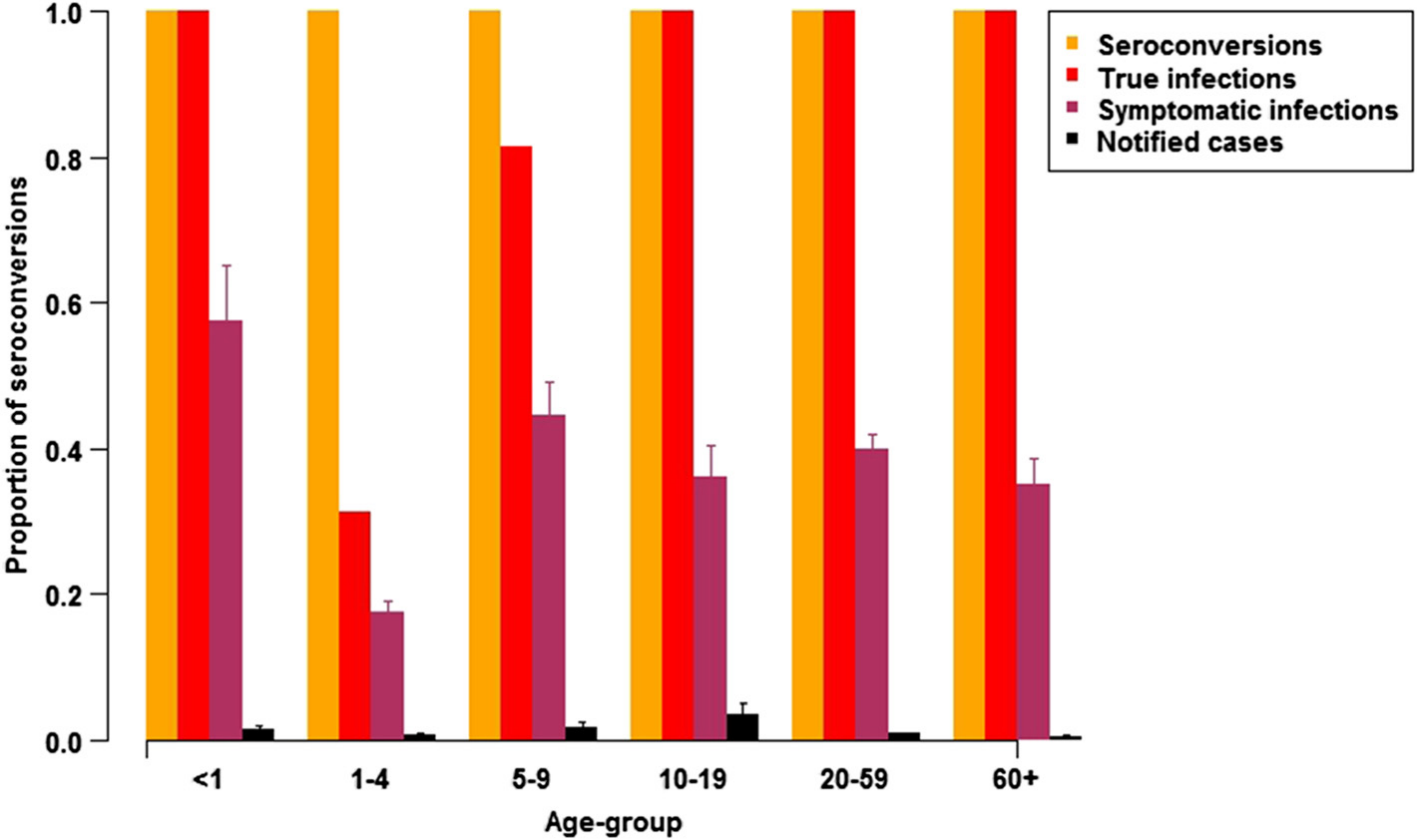
The ‘surveillance pyramid’ (proportions of all seroconversions that are true infections (i.e., not vaccination-related), symptomatic infections, and that were notified) aggregated over the period 2005–2011, and plotted separately for each age-group. ‘True infections’ indicate seroconversions adjusted for vaccination-related high titres. Capped segments indicate 95 % credible intervals

The overall SI incidence rate was estimated at 128 per 10,000 population in 2005, decreasing to 107 per 10,000 in 2011. Temporal trends in SI incidence rates over the study period varied by age; annual SI incidence rates significantly decreased by 7.8 %, 24.3 % per year and 15.9 % per year on average, for the <1 years, 1–4 years, and 5–9 years age-groups respectively, but increased by 1.7 % per year on average for the 20–59 years age-group (Table 4 and Fig. 3).

**Table 4:** Other estimated quantities

Age-group	Average annual percentage change in SI incidence (95 % CI)	MF (95 % CrI)
<1 year	−7.8 %	41
	(−8.3, −7.4 %)	(23–66)
1–4	−24.3 %	25
	(−24.7, −23.9 %)	(17–34)
5–9	−15.9 %	26
	(−16.2, −15.7 %)	(16–39)
10–19	−0.4 %	10
	(−0.6, −0.1 %)	(6–15)
20–59	1.7 %	47
	(1.6, 1.8 %)	(37–59)
60+	−1.8 %	69
	(−1.9, −1.6 %)	(49–96)

Average annual percentage change in SI rates (posterior median estimate of SI cases per 10,000 persons), and posterior median estimates of the age-group specific multiplication factor (MF) for converting notified cases to the estimated number of SI cases. 95 % credible intervals (CrI) are in parentheses. Estimates for the youngest three age-groups are potentially affected by vaccination-related seroconversions

**Fig. 4:**
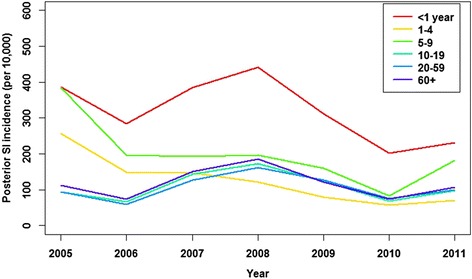
Estimated incidence rate (cases per 10,000 persons) of symptomatic pertussis infection over the period 2005–2011, plotted separately for each age-group. Posterior median estimates only are shown

Multiplication factors (MFs) to convert notified case numbers to the true number of SI cases were derived as (1/*d*_*a,SI*_), for each age-group separately (Table 4), and so were not assumed to vary smoothly with age. Under-estimation was most pronounced for the 60+ years age-group (Fig. 4), with an MF of 69 (95 % CrI: 49–96).

We additionally estimated the extent to which the Markov model-estimated proportions of vaccination-related high IgG-Ptx titres for the 1–4 and 5–9 years age-groups were influenced by the value assumed for the waning rate parameter. When specifying either a 10 % higher or a 10 % lower value for this parameter, there were only small differences in posterior median SI incidence rate for these two age-groups (Additional file 1: Table S2), which indicates that our prinicipal results were not unduly sensitive to one assumption inherent in the estimation of vaccination-related seroconversions. Finally, prior and posterior distributions for the parameter *c*_*a,SI|I*_ are graphically compared in (Additional file 1: Figure S2).

## Discussion

Using evidence-synthesis methods, we estimated the incidence of symptomatic pertussis infection over the period 2005–2011 in the Netherlands. Aggregating over age, the estimated SI incidence rate ranged from a low of 76 per 10,000 (95 % CrI: 64–89) in 2010 to a high of 173 (95 % CrI: 147–203) in 2008.

Decreasing trends in estimated SI incidence rates were apparent over study period for all age-groups except for the 20–59 years age-group; the greatest annual average decreases were observed for the 1–4 years and 5–9 years age-groups (−24.3 %, and −15.9 % per annum, respectively), consistent with the replacement of the whole cell by the acellular vaccine (i.e., improving vaccine effectiveness) in the infant vaccination in 2005 (and in the booster since 2001) [1, 2]. Previous research has shown that within the period 1996–2011, a declining trend in notification rates was associated with vaccination measures for only those age-groups eligible for vaccination, i.e., up to ages 4–6 years [2]. Our SI incidence rate trends largely concur; however, the weak decreasing trend in SI incidence rate (−1.8 %) that we estimated for the 60+ years age-group appears inconsistent with reported increases in seroprevalence in >9 year-olds over time (between 1995/6 and 2006/7 [1]). In general, temporal trends in the estimated SI incidence rate were consistent with trends in case notification rates over the period 2005–2011, including a rise in cases observed in 2007–2008 and again in 2011 [2]. The age-dependent pattern of SI trends suggests that although the acellular vaccine has prevented more infections in children, circulation has not been greatly affected.

We could also estimate the extent to which the national notification system under-represented the numbers of individuals with SI. The derived multiplication factors varied with age-group, from 10 (95 % CrI: 6–15) to 69 (95 % CrI: 49–96), for the age-groups 10–19 years and 60+ years, respectively. This age-dependent variation in the estimated degree of under-notification may be due to (a combination of) age differences in disease severity, the likelihood of contacting primary health care, diagnostic practice, and/or reporting bias. Although the estimated MF for the <1 year age-group was also high (41; 95 % CrI: 23–66), suggesting a large number of unreported symptomatic infections in young infants, no correction for vaccination-related high IgG-Ptx titre was attempted for this age-group.

Previous research in the Netherlands has estimated the incidence of either symptomatic or asymptomatic infection from the prevalence of serological markers. Based on an anti-Ptx IgG concentration threshold of 125 EU/ml, de Greeff and colleagues [1] estimated that 3.4 % (95 % CI: 2.8–3.9 %) of the Dutch population aged >9 years had had a pertussis infection within the six months prior to their PIENTER-2 sample date in 2006/2007. Synthesising all the evidence available, we estimated the posterior median incidence of seroconversion (i.e., both symptomatic and asymptomatic infection) for persons aged 10+ years at 240,100 and 508,400 persons in 2006 and 2007, respectively, which corresponds to 1.7 % (95 % CI 1.4–1.9 %) and 3.5 % (95 % CI 3.1–4.0 %) of the population aged 10+ years in each of these two years, respectively. These estimates differ from the priors specified for the seroconversion probability, because the posteriors for these parameters are also informed by indirect evidence from the rest of the model.

There has been substantial recent progress in using mathematical modelling methods to understand aspects of the epidemiology of pertussis infection [19–22], including how natural immunity may underlie long-term trends [19], and how changes in the duration of vaccine-induced and natural immunity can account for trends in incidence [20]. Such dynamic modelling approaches are useful both for explaining historical incidence patterns and for forecasting. Unlike these approaches, our study derives the national-level incidence of symptomatic pertussis infection using statistical modelling. Advantages of the Bayesian evidence synthesis approach adopted here include provision of a coherent, flexible framework in which diverse sources of information can be combined and the correct propagation of uncertainty associated with all model parameters to the final estimates.

Limitations to the current approach should also be noted. First, the validity of the current SI estimates is dependent on the assumptions made when specifying relationships between evidence sources and on the quality (representativeness and bias) of the observed data and prior information. For instance, if bias is present in the data sources used to inform the symptomatic proportion parameters, SI incidence would be affected. Symptomatic proportions for several age-groups were derived from PIENTER-2, which may biased downward because recall of coughing symptoms in the previous year was required. In contrast, the values for this parameter derived from the BINKI study may be upwardly biased, because household contacts of infected infants may have more severe disease compared with community study participants.

A second limitation concerns the interpretation of seroconversion. For children under 5 years of age, of whom more than 90 % will have been vaccinated [12], a single sample with a high IgG-Ptx titre cannot distinguish between infection and previous vaccination. The Dutch National Immunisation Programme recommendations stipulate administration of a pertussis booster at four years of age; given that high vaccination-related IgG-Ptx levels wane rapidly [23, 24], a proportion of high IgG-Ptx titres in 5-year-olds and a smaller proportion in 6 through 9 year olds are likely attributable to the 4-years booster. Although we corrected PIENTER-2 seroprevalence in the 1–4 years and 5–9 years age-groups for the model-estimated proportion of vaccination-related high titres (Additional file 1: Table S1), our estimates should still be interpreted with caution, as this adjustment depends on the model parameters and other assumptions. It was not feasible to adjust the <1 years age-group due to the granularity of the seroprevalence and vaccination coverage data.

Pertussis incidence displays long-term periodicity [25]. We recognise that if a different study period was chosen – for instance if the outbreak year 2012 had been included in the analysis – reported trends in annual SI incidence, and their interpretation, may differ.

Finally, the degree of under-representation of the number of ‘true’ SI cases by the statutory notification system depends on several factors, such as health-care seeking behaviour, diagnostic accuracy, reporting bias, and severity of disease. All of these factors are plausibly dependent on age, but could also vary over time. Although we have estimated MFs separately by age-group, we assumed that MFs were constant over time to aid identifiability of the statistical model. For the same reason, we constrained the probability of developing symptoms to be time-independent. If symptom severity is associated with temporal changes in transmission [26], then model outcomes may be over-simplified.

## Conclusions

In summary, by applying Bayesian evidence synthesis methodology to a variety of national data sources, we have derived robust age group-specific estimates of the incidence of symptomatic pertussis infection in the Netherlands. This information is essential for determining the pertussis disease burden, and together with modelling and other studies [27], can assist in informing policy decisions regarding the design and improvement of preventive measures.

## Availability of data and materials

For access to data used in this study that are not publicly available, please get in contact with the first author.

## Additional file

Additional file 1:
**OpenBUGS code, description of the Markov model for estimating vaccination-related high titres, and additional figures.** (PDF 379 kb)

## Abbreviations

SI: Symptomatic infected
CrI: Credible interval
MPES: Multi-parameter evidence synthesis
SIAR: Symptomatic infection attack rate
MF: Multiplication factor
