# Supplementary material for: An evidence synthesis approach to estimating the incidence of symptomatic pertussis infection in the Netherlands, 2005–2011
Source: BMC Infect Dis. 2015 Dec 29;15:588. doi: 10.1186/s12879-015-1324-y (PMC4696101; doi:10.1186/s12879-015-1324-y)
Supplement: Additional file 1: — OpenBUGS code, description of the Markov model for estimating vaccination-related high titres, and additional figures. (PDF 379 kb) [file 12879_2015_1324_MOESM1_ESM.pdf]

SUPPLEMENTARY MATERIALS

To accompany the article in *BMC Infectious Diseases*:

Title: An evidence synthesis approach to estimating the incidence of symptomatic pertussis infection in the Netherlands, 2005–2011

Authors: Scott A. McDonald, Peter Teunis, Nicoline van der Maas, Sabine de Greeff, Hester de Melker, Mirjam E. Kretzschmar

Corresponding author: Scott A. McDonald, Centre for Infectious Disease Control
National Institute for Public Health and the Environment. E-mail: scott.mcdonald@rivm.nl

In this supplementary materials document, OpenBUGS code for the model is provided, further information is supplied regarding the Markov model developed to adjust seroconversion probabilities for the 1-4 years and 5-9 years age-groups for vaccination-related high Ptx-IgG titres, and a graphical comparison of the prior and posterior distributions for the probability of being symptomatic given infection is made.

OpenBUGS code

```
model {

   ## Vague Beta prior on d_SI and random-walk prior on c_S|Pop
   ## NG is number of age-groups; NY is number of years
   for (a in 1:NG) {
     d.SI[a] ~ dbeta(1,1)     # Detection probability

     for (y in 1:NY) {        # Convert from logit scale
       logit(c.SgivenPop[a, y]) <- logitc.SgivenPop[a,y]
     }
     logitc.SgivenPop[a, 1] ~ dnorm(0,0.0001)

     tau[a] ~ dgamma(0.001,0.001)
     for (y in 2:NY) {
       logitc.SgivenPop[a, y] ~ dnorm(logitc.SgivenPop[a, y-1],tau[a])
     }
   }

   ## Beta priors on c_SI|I: PIENTER-2 (ages 10-60+) and expert opinion(<1 yr)
   ## Parameters of beta prior (a and b) supplied as Prop.Sympt.a and Prop.Sympt.b
   c.SIgivenI[1] ~ dbeta(Prop.Sympt.a[1],Prop.Sympt.b[1])
   c.SIgivenI[4] ~ dbeta(Prop.Sympt.a[4],Prop.Sympt.b[4])
   c.SIgivenI[5] ~ dbeta(Prop.Sympt.a[5],Prop.Sympt.b[5])
   c.SIgivenI[6] ~ dbeta(Prop.Sympt.a[6],Prop.Sympt.b[6])

   ## Vague Beta priors on c_SI|I for 1-4 and 5-9 yrs
   for (a in 2:3) {
     c.SIgivenI[a] ~ dbeta(1, 1)
   }

   ## Update c_SI|I with data: BINKI (age-groups 1-4 through 20-59 yrs only)
   ## Data supplied as positives (y.Obs.Prop.Sympt) and total (n.Obs.Prop.Sympt)
   for (a in 2:5) {
     y.Obs.Prop.Sympt[a] ~ dbin(c.SIgivenI[a],n.Obs.Prop.Sympt[a])
   }

   ## Loop for likelihood and functional expressions
   ## Seroconversion data inform c_S|Pop for year=3 (2007) only:
   for (a in 1:NG) {
     y.Obs.Prop.Pienter2[a, 3] ~ dbin(c.SgivenPop[a, 3],n.Obs.Prop.Pienter2[a, 3])

     MF[a] <- ( 1/d.SI[a] )                    # Define multiplication factor

     for (y in 1:NY) {
       y.Obs.Num.Notif[a, y] ~ dbin(d.SI[a],n.SI[a, y])
       SIAR[a, y] <- (c.SIgivenI[a]*c.IgivenPop[a, y])   # Define SI attack rate

       # Adjust c_I|Pop for vaccination-related seroconversions
       c.IgivenPop[a,y] <- (c.SgivenPop[a, y] * (1 - adjVacc[a]))

       # Numbers in S, I and SI subpopulations
       n.S[a, y] <- (c.SgivenPop[a, y] * n.Pop[a, y])
       n.I[a, y] <- (c.IgivenPop[a, y] * n.Pop[a, y])
       n.SI[a, y] <- (c.SIgivenI[a] * n.I[a, y])
     }
   }

   ## Aggregated-over-age values in index (NG+1)
   for (y in 1:NY) {
     n.I[(NG+1), y] <- sum(n.I[1:NG, y])
     n.SI[(NG+1), y] <- sum(n.SI[1:NG, y])
     SIAR[(NG+1), y] <- n.SI[(NG+1), y]/sum(n.Pop[1:NG, y])
   }
}
```

Adjustment for vaccination-related high Ptx-IgG titres

In the following, we describe how the seroconversion probabilities for the age-groups 1-4 and 5-9 years were adjusted, to account for the proportion of each age-group with a high Ptx-IgG titre attributable to previous vaccination. We constructed a simple Markov model of the waning titre expected using the recommended immunization schedule: a series of four infant vaccinations is administered at 2, 3, 4, and 11 months of age, with a booster vaccination given at four years of age [1, 2].

Fig. S1 shows the multi-state Markov model. The flow of children aged one through nine years, over the period 1995 through 2011, was modelled. One-year olds enter one of two compartments: *Vaccinated (Infant)*, with probability β; or *Unvaccinated* with probability $(1 - \beta)$. β is the vaccination coverage (i.e., percentage with base immunity) for the relevant simulation year. For convenience, immune response to the vaccine is assumed to result in an Ptx-IgG titre of ≥125 EU/ml. Titres are then assumed to wane with an annual probability of 69%. This annual transition probability, P_{γ}, was derived from predictions of a statistical model fitted to longitudinal data on natural pertussis infection [3]. Specifically, the transition probability P_{γ} approximates the rate by which the fraction of infected persons with titre ≥125 declines as a function of time since infection. When children turn four years of age, they receive a booster vaccination; thus children of this age only who are in the *Vaccinated (Infant)* and *Titre < 125 EU/ml* compartments can move to the *Vaccinated (Booster)* compartment with probability P_{φ}, where is P_{φ} represents the booster vaccination coverage for the relevant year.

The simulation provided estimates of the proportion of children aged one through nine years with a vaccination-related Ptx-IgG titre ≥125 EU/nl, for the years 2005 through 2011 (Table S1). There was little variation across time, except for the oldest ages in 2005 and 2006, which is due to the four-year booster being introduced in 2002 (e.g., children who were eight or nine years old in 2005 would have been too old to receive the booster in 2002) . For use in the evidence synthesis model, we estimated separate proportions with Ptx-IgG titre ≥125 due to vaccination for the 1-4 years and 5-9 years age-groups, by averaging together the values for the individual ages in each age-group for the simulation, and then averaging over years. The

resulting estimated proportions of vaccination-related high titres are 0.687 and 0.185 for age-groups 1-4 and 5-9 years, respectively.

To establish the extent to which the model-estimated proportions of vaccination-related high titres are sensitive to the value used for the annual transition probability (P_γ) we re-ran the Markov model using 10% higher and 10% lower values of P_γ, and derived the estimated proportions of vaccination-related high titres for the age-groups 1-4 and 5-9 years, as before (see Table S2). Then, we repeated the evidence synthesis using these modified values of *propVaccRel*, and report the posterior median symptomatic infection (SI) incidence rates (per 10,000) for these two age groups, comparing with the posterior median SI incidence rates obtained using the baseline value for P_γ (Table S2). There were only small differences apparent; depending on year, there was a maximum 3% increase in estimated SI incidence rate if the higher value for P_γ was adopted, and up to a 6% decrease if the lower value was used.

Table S1. Modelled percentage of children with Ptx-IgG titre ≥125 EU/ml due to vaccination, 2005-2011, as estimated using vaccination uptake data and model-derived waning antibody rates.

	Calendar year						
Age (years)	2005	2006	2007	2008	2009	2010	2011
1	94.0	94.5	95.2	95.0	95.4	95.4	95.5
2	57.1	57.0	57.2	57.5	57.4	57.5	57.5
3	41.3	41.0	40.9	41.0	41.1	41.1	41.2
4	82.6	83.2	82.9	82.7	83.0	83.4	83.4
5	33.6	33.7	33.8	33.6	33.6	33.7	33.8
6	19.0	19.4	19.3	19.2	19.1	19.1	19.1
7	14.2	14.1	14.6	14.4	14.3	14.2	14.2
8	17.0	12.2	12.0	12.5	12.3	12.2	12.1
9	15.2	15.2	11.0	10.9	11.3	11.1	11.1

Table S2. Results of sensitivity analysis to baseline and 10% lower and 10% higher values assumed for the annual transition probability of waning high Ptx-IgG titre (P_γ). *propVaccRel* refers to the model-derived proportion of vaccination-related high titres.

		Posterior median SI incidence rate (per 10,000)						
P_γ	*propVaccRel*	2005	2006	2007	2008	2009	2010	2011
Age-group 1-4 years								
69.0%	0.687	257	149	147	121	79	57	70
62.7%	0.704	243	141	138	114	74	54	66
75.9%	0.678	264	153	151	124	81	58	72
Age-group 5-9 years								
69.0%	0.185	386	196	193	197	160	82	182
62.7%	0.206	377	191	188	192	156	81	178
75.9%	0.164	395	201	197	202	164	84	187

Fig. S1. Multi-state Markov model for pertussis vaccination and waning anti-Ptx-IgG titre.

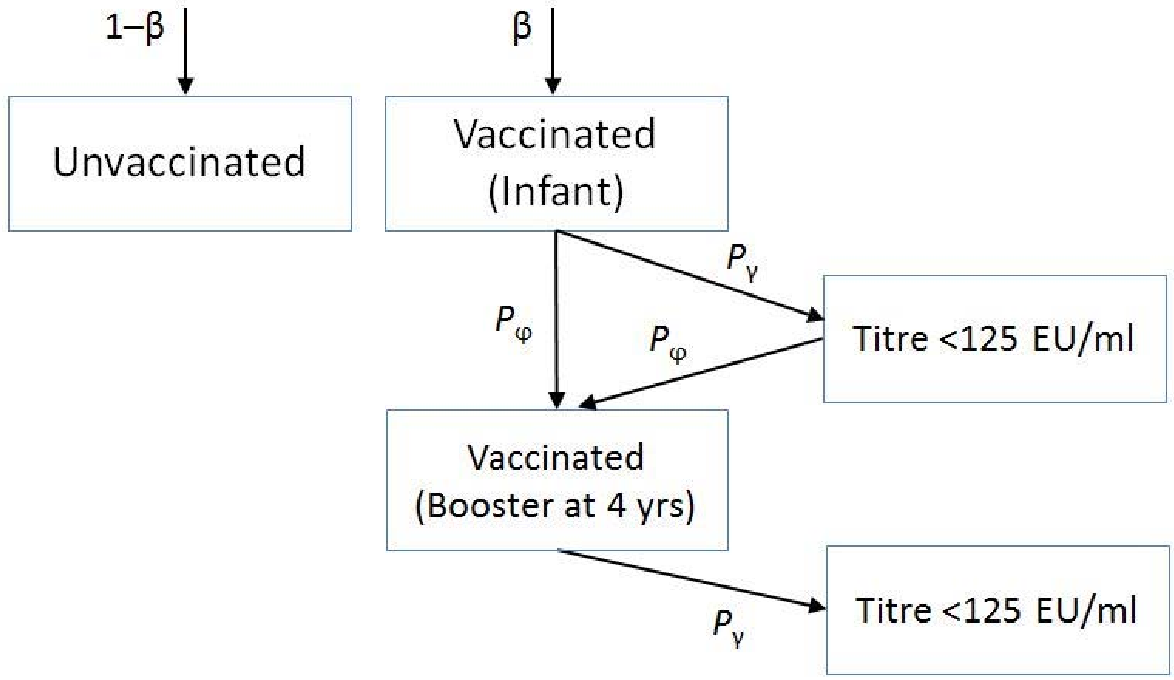

Fig. S2. Prior (i.e., reflecting both PIENTER-2 and BINKI data) and posterior distributions for the parameter $c_{a,SI|I}$.

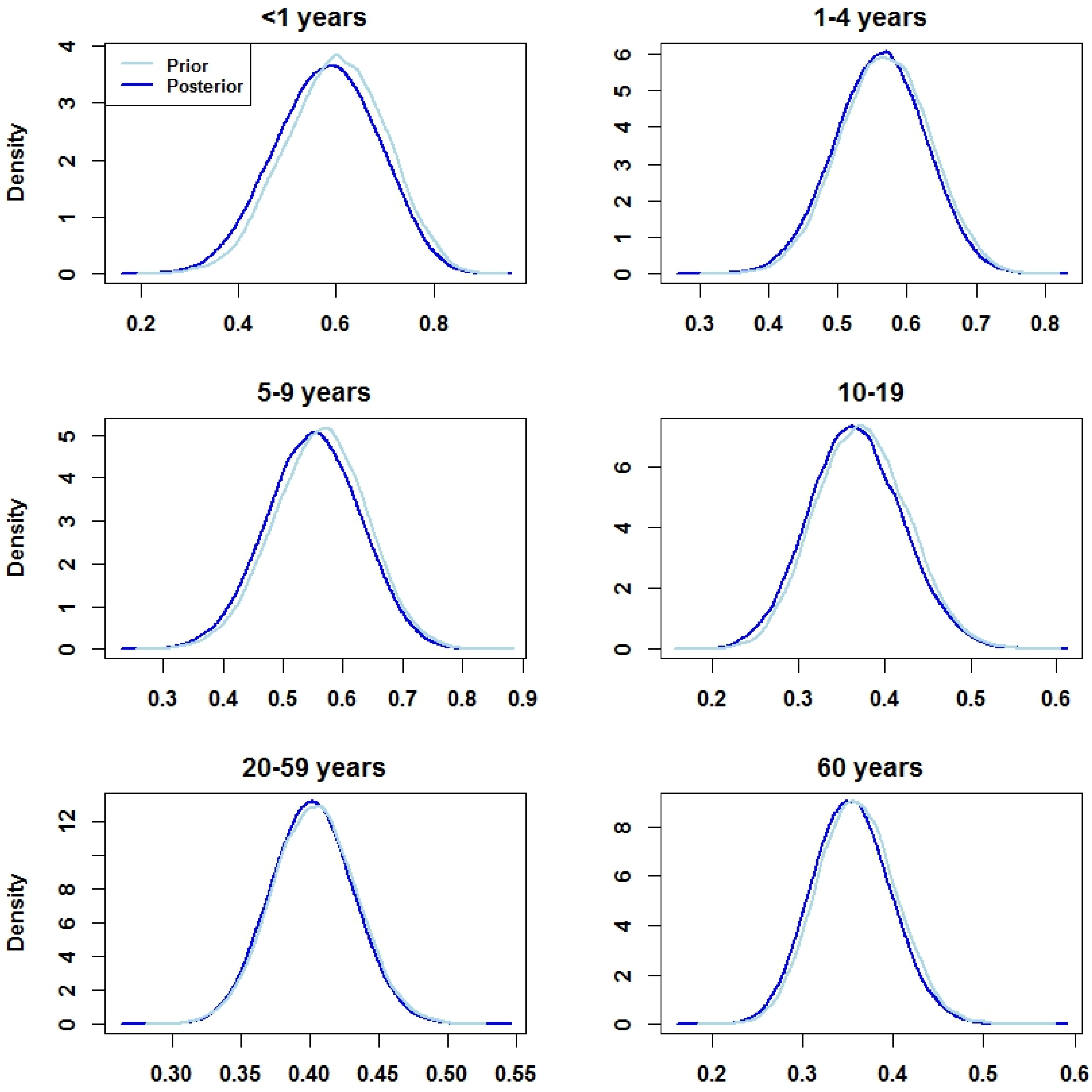

References

1. van Lier EA, Oomen PJ, Oostenbrug MWM, Zwakhals SLN, Drijfhout IH, de Hoogh PAAM et al. Vaccination coverage of the National Immunization Programme in the Netherlands, 2006–2008. Bilthoven: RIVM; 2008 (RIVM report 210021007).

2. van Lier A, P.J. Oomen, H. Giesbers, M.A.E. Conyn-van Spaendonck, I.H. Drijfhout, I.F. Zonnenberg-Hoff, H.E. de Melker. Vaccination coverage of the National Immunization Programme in the Netherlands: Report year 2014. Bilthoven: RIVM; 2014 (RIVM report 150202003).

3. de Graaf, W. F., Kretzschmar, M. E. E., Teunis, P. F. M., & Diekmann, O. (2014). A two-phase within-host model for immune response and its application to serological profiles of pertussis. Epidemics, 9, 1-7.
